# Virtual Reality Reduces Perioperative Anxiety During Cardiac Device Implantation: A Randomized Trial

**DOI:** 10.1016/j.jacadv.2026.103267

**Published:** 2026-09-23

**Authors:** Amirhossein Givan, Jan-Eric Bohnen, Christiane Jungen, Tienush Rassaf, Shibu Mathew, Amir Abbas Mahabadi, Muhammed Kurt

**Affiliations:** Department of Cardiology and Vascular Medicine, West German Heart and Vascular Center Essen, University of Duisburg-Essen, Essen, Germany

**Keywords:** cardiac implantable electronic device, conscious sedation, midazolam, nonpharmacologic intervention, perioperative anxiety, randomized controlled trial, virtual reality

**Figure:**
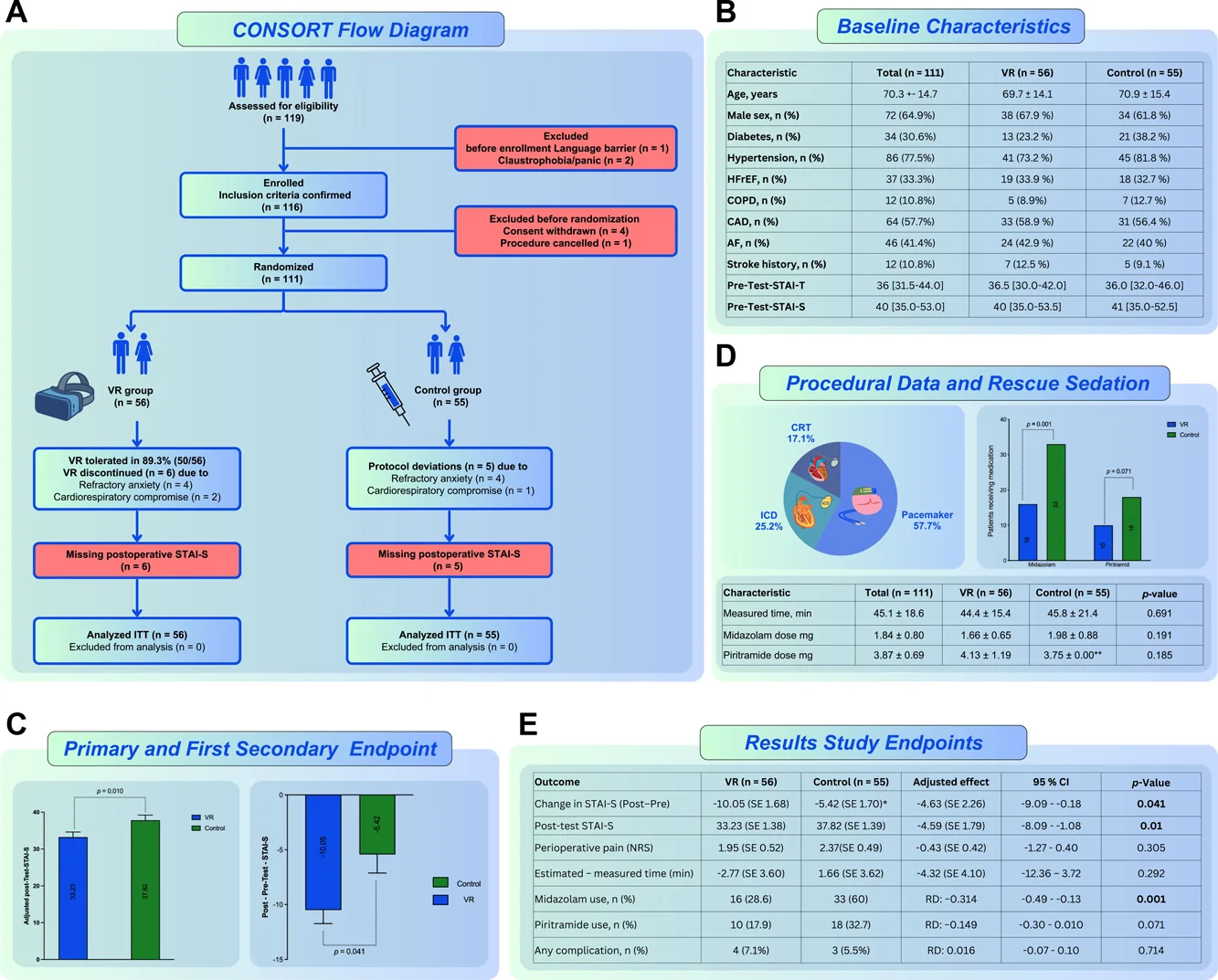

**What is the clinical question being addressed?**

Does intraoperative use of VR reduce perioperative anxiety in patients undergoing cardiac implantable electronic device implantation under local anesthesia with on-demand conscious sedation?

**What is the main finding?**

VR significantly reduced perioperative state anxiety, the primary endpoint (*P* = 0.041). As a secondary exploratory outcome, rescue midazolam administration was lower in the VR group (28.6% vs 60.0%).

The contemporary anesthetic approach to cardiac implantable electronic device (CIED) implantation has evolved toward local anesthesia combined with on-demand intravenous conscious sedation.^1^ However, administration of sedative medications is often associated with cardiopulmonary side effects, particularly in elderly patients and those with significant comorbidities such as heart failure with reduced ejection fraction or chronic obstructive pulmonary disease.^2^ Virtual reality (VR) has emerged as a nonpharmacologic distraction modality that may attenuate perioperative anxiety through immersive audiovisual engagement redirecting attention away from procedural stimuli, and it has been shown to reduce perioperative anxiety in selected cardiovascular interventions performed under local anesthesia, including transcatheter aortic valve implantation.^3^ To date, prospective randomized data evaluating VR throughout the entire CIED implantation procedure with a validated standardized anxiety questionnaire are scarce.

This prospective, randomized, open-label, single-center controlled trial was conducted at the West German Heart and Vascular Center, Essen, Germany, between January 2024 and May 2025 (NCT07250282). The study protocol was approved by the ethics commission of the University of Duisburg-Essen on September 27, 2023 (approval number: 23-11442-BO), before enrollment of the first participant. The study was performed in accordance with the Declaration of Helsinki and reported following the Consolidated Standards of Reporting Trials guidelines. Written informed consent was obtained from all participants. Patients aged ≥18 years with an indication for de novo CIED implantation or system upgrade were eligible. Exclusion criteria comprised severe panic disorder or claustrophobia and significant language barriers precluding informed consent or cooperation.

An a priori calculation assuming a standardized effect size of Cohen's d = 0.55, a conservative estimate derived from prior interventional cardiology studies using the State-Trait Anxiety Inventory state subscale (STAI-S) under local anesthesia,^3^ indicated a minimum of 106 participants (53 per group) for 80% power at two-sided α = 0.05 (G∗Power 3.1.9.6). Consecutive eligible patients were enrolled until this threshold was exceeded, allowing for anticipated missing outcome data. A total of 111 patients were randomized 1:1 via a computer-generated sequence, using simple randomization with stratification by device type, to VR plus standard care (n = 56) or standard care alone (n = 55). In the VR group, patients wore a Conformité Européenne-certified 2D VR headset displaying self-selected videos (HappyMed) throughout the procedure. The system delivered nonstereoscopic immersive audiovisual content via a head-mounted display without positional tracking. Available content was licensed for clinical use and comprised meditation, feature films, filmed theater, travel documentaries, and nature programs. Standard care, applied uniformly to both groups, comprised subcutaneous lidocaine 1% (400 mg in 40 mL) at the incision site with a prespecified threshold-based rescue protocol. Midazolam 1 mg intravenously was administered for anxiety scored ≥5 on a numeric rating scale (NRS), and piritramide 3.75 mg for pain scored ≥5 on the NRS, with additional doses for persistent symptoms. Continuous electrocardiographic and pulse-oximetric monitoring was maintained throughout, with oxygen delivered using a nasal cannula and escalated to a face mask at higher flow rates if required. Headset use did not alter this protocol. The primary endpoint was the between-group difference in change in STAI-S score from preoperative baseline to immediate postoperative assessment, analyzed under the intention-to-treat framework using a mixed model for repeated measures across the 2 assessment timepoints, with a group-by-time interaction term. Secondary endpoints included baseline-adjusted immediate postoperative STAI-S, perioperative pain (NRS), estimated vs actual procedure time, and the rates of conscious sedation and analgesia administration. Procedure-related complications were assessed through 6 months. Sensitivity analyses included complete-case analysis (Mann–Whitney U test) and pattern-mixture missing-not-at-random imputation with delta adjustment for both primary and secondary endpoints with missing data.

Baseline clinical and demographic characteristics were balanced between groups (mean age: 70.3 ± 14.7 years; 64.9% male; hypertension 77.5%; coronary artery disease 57.7%; atrial fibrillation 41.4%). Devices implanted were pacemakers (57.7%), implantable cardioverter-defibrillators (25.2%), and cardiac resynchronization therapy (17.1%). Baseline state and trait anxiety were comparable between groups. Values are reported as median and IQR. Pretest State-Trait Anxiety Inventory trait subscale was 36.5 (30.0-42.0) in the VR group vs 36.0 (32.0-46.0) in controls, and pretest STAI-S was 40 (35.0-53.5) vs 41 (35.0-52.5). Moreover, length of hospital stay, intensive care unit admission rates, and procedure duration did not differ between groups. Procedural success was 100%. No cybersickness symptoms were reported. The VR headset was tolerated in 89.3% of patients. Intraoperative removal was necessary in 6 patients (4 for refractory anxiety requiring sedation escalation, and 2 for cardiorespiratory compromise unrelated to the VR headset). Postoperative STAI-S could not be obtained in 11 patients (6 VR and 5 control) because of intraoperative clinical adverse events. These were retained in the intention-to-treat framework with appropriate handling of missing data.

For the primary endpoint, the adjusted between-group difference in STAI-S change from preoperative baseline to immediate postoperative assessment was −4.63 points (95% CI: −9.09 to −0.18; *P* = 0.041), favoring VR (Figure 1C). Among secondary endpoints, baseline-adjusted immediate postoperative STAI-S was also significantly lower in the VR group (estimated marginal mean: 33.23 vs 37.82; adjusted difference: 4.59, 95% CI: −8.09 to −1.08; *P* = 0.010; Figure 1C), reinforcing the primary finding. Perioperative pain was low and not significantly different between groups (adjusted difference: −0.43, 95% CI: −1.27 to 0.40; *P* = 0.30), nor was estimated vs actual procedure time (adjusted mean difference: −4.32 minutes, 95% CI: −12.36 to 3.72; *P* = 0.29). As a further secondary finding, the proportion of patients receiving midazolam was significantly lower in the VR group (28.6% vs 60.0%; risk difference: −0.314; 95% CI: −0.49 to −0.13; *P* = 0.001), corresponding to a number needed to treat of 4. Piritramide administration rates were not statistically different (17.9% vs 32.7%; *P* = 0.071). Cumulative doses of midazolam and piritramide were comparable among recipients. No significant difference in procedure-related complications was observed through 6 months (VR 7.1% vs control 5.5%; *P* = 0.71). Complications in the VR group comprised pericardial tamponade (n = 1), lead dislodgement (n = 1), lead perforation (n = 1), and pocket hematoma (n = 1), and in the control group, they include lead dislodgement (n = 1), lead-related endocarditis (n = 1), and pneumothorax (n = 1). Given the low event frequency, this 6-month comparison is descriptive only. Sensitivity analyses consistently supported the primary and secondary findings.

**Figure 1:**
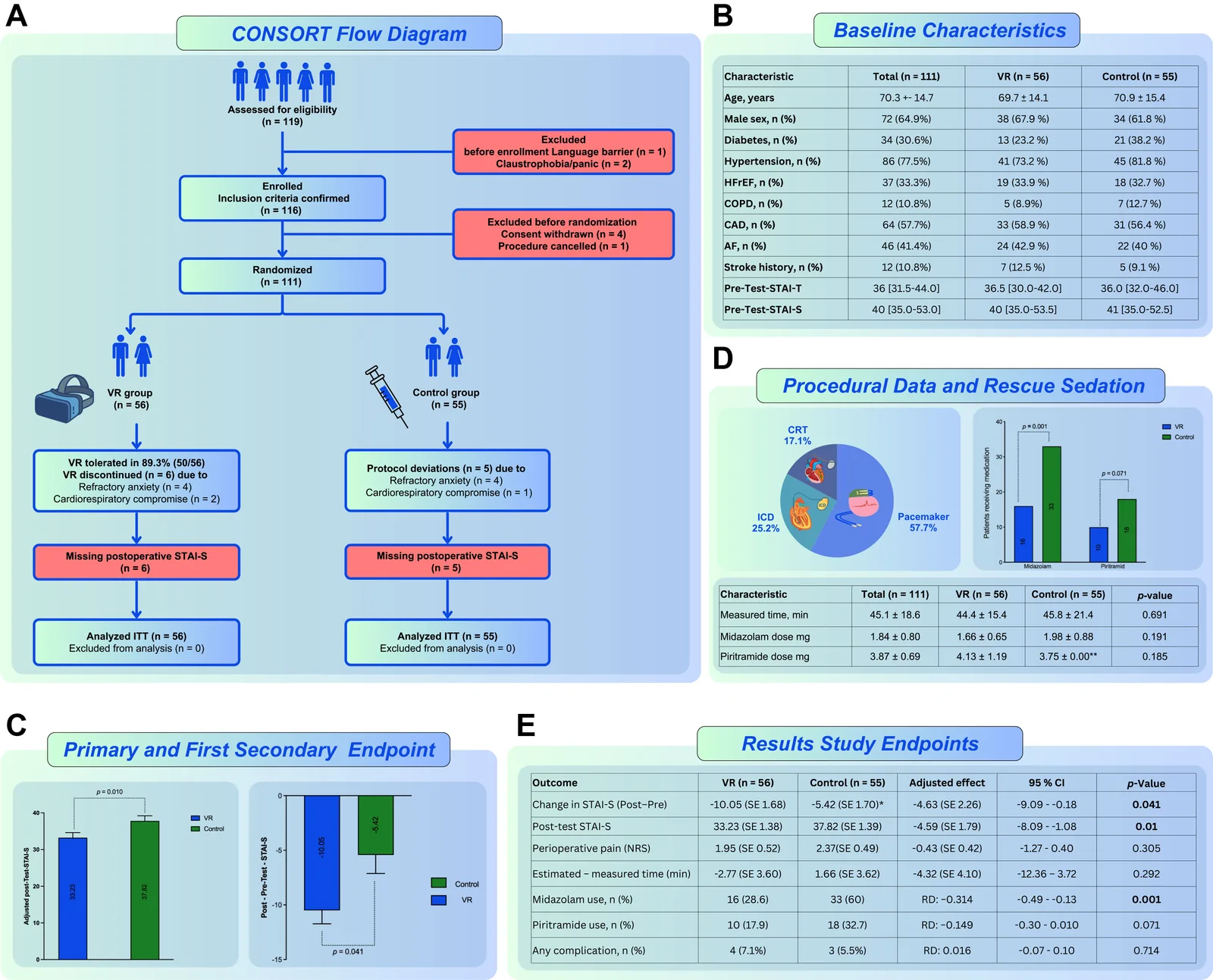
Virtual Reality for Anxiety Reduction During Cardiac Device Implantation All analyses followed the intention-to-treat principle. (A) CONSORT flow diagram; the headset was removed intraoperatively in 6 patients: 4 for refractory anxiety and 2 for hemodynamic or respiratory compromise. (B) Baseline characteristics, shown as mean ± SD or median [IQR]. (C) Primary endpoint (between-group difference in change in STAI-S from baseline to postoperative assessment) and first secondary endpoint (adjusted post-test STAI-S). (D) Procedural data and rescue sedation, including cumulative midazolam and piritramide doses. (E) Study endpoints with procedure-related complications through 6 months, descriptive only. Bars show estimated marginal means; error bars represent standard errors. AF = atrial fibrillation; CAD = coronary artery disease; CIED = cardiac implantable electronic device; CONSORT = Consolidated Standards of Reporting Trials; COPD = chronic obstructive pulmonary disease; CRT = cardiac resynchronization therapy; HFrEF = heart failure with reduced ejection fraction; ICD = implantable cardioverter-defibrillator; ITT = intention-to-treat; NRS = numeric rating scale; RD = risk difference; SE = standard error; STAI-S and STAI-T = State-Trait Anxiety Inventory, State and Trait subscales; VR = virtual reality.

The primary finding was a reduction in perioperative anxiety with the intraoperative use of VR during CIED implantation. A secondary finding was the lower rate of midazolam administration in the VR group, reflecting fewer patients reaching the prespecified anxiety threshold. The selective effect on sedation without an effect on opioid analgesia supports a specific anxiolytic action of VR. Headset removal for refractory anxiety in 4 patients indicates that VR does not replace pharmacologic rescue in all cases, requiring a protocol that permits immediate removal and sedation escalation. A recent state-of-the-art review concluded that evidence across cardiac surgery and interventional cardiology supports a potential anxiolytic benefit of VR, while highlighting the need for adequately powered randomized trials using standardized outcome measures.^4^ To date, only one prior randomized study has evaluated VR during pacemaker and implantable cardioverter-defibrillator implantation, assessing anxiety and pain using NRS at discrete procedural timepoints.^5^ Squara et al^5^ found reduced pain and anxiety at axillary vein puncture but not during pocket creation, with lower morphine consumption. Their trial used a nonstereoscopic, noninteractive 360° video with recorded hypnotic suggestion, applied preoperatively and worn throughout, whereas the present study used 2D video applied intraoperatively. Notably, in that trial, intravenous paracetamol was administered to all patients preoperatively, making pain rather than anxiety the dominant unmet need, whereas the present trial, without routine preoperative analgesia, addressed anxiety. Accordingly, the principal effect of VR was a reduction in anxiety-driven sedation rather than opioid consumption. However, since sedation was more frequent in the control group (60.0% vs 28.6%), the anxiolytic and amnestic effects of midazolam would attenuate rather than amplify the between-group difference, so the true effect is likely larger than the observed 4.63 points, which nonetheless reached significance. Methodological strengths include prospective enrollment, the full spectrum of CIED procedures, the validated STAI instrument, an intention-to-treat framework, and a prespecified rescue protocol applied uniformly to both groups, reducing performance bias inherent to open-label trials. The secondary sedation-sparing finding could be of particular clinical relevance in the typical CIED population, in whom intravenous sedation carries heightened cardiopulmonary risk. Limitations include the single-center design, the absence of blinding due to the visible nature of the intervention, and the missing postoperative anxiety data despite imputation. Content was self-selected and therefore heterogeneous in genre, arousal level, and personal relevance, and baseline anxiolytic or psychotropic medication use was not recorded as a covariate. As no prior STAI-based data existed in this population, the assumed effect size was derived from methodologically comparable trials, which may have led to an overestimate of the achieved power. In conclusion, the intraoperative use of VR is a feasible, safe, and effective nonpharmacologic adjunct that significantly reduces perioperative anxiety during CIED implantation, with reduced conscious sedation as a secondary finding. Confirmation in multicenter, ideally blinded randomized trials is warranted.

## Funding support and author disclosures

The authors acknowledge support by the Open Access Publication Fund of the University of Duisburg-Essen, Essen, Germany. Dr Jungen is supported by the BMFTR (Federal Ministry of Research, Technology and Space), Advanced Clinician Scientist Programme UMEA2 (01EO2104). Dr Rassaf has received honoraria from Daiichi Sankyo, Alnylam, and Bristol Myers Squibb for lectures. All other authors have reported that they have no relationships relevant to the contents of this paper to disclose.
